# Supplementary material for: Waterproof and ultraflexible organic photovoltaics with improved interface adhesion
Source: Nat Commun. 2024 Feb 1;15:681. doi: 10.1038/s41467-024-44878-z (PMC10834485; doi:10.1038/s41467-024-44878-z)
Supplement: Supplementary file 1 — Supplementary Information [file 41467_2024_44878_MOESM1_ESM.pdf]

Supplementary Information

Waterproof and ultraflexible organic photovoltaics with improved interface adhesion

Sixing Xiong[1], Kenjiro Fukuda[1, 2*], Kyohei Nakano[1], Shinyoung Lee[1], Yutaro Sumi[3], Masahito Takakuwa[3,4], Daishi Inoue[1], Daisuke Hashizume[1], Baocai Du[1,3], Tomoyuki Yokota[3,4], Yinhua Zhou[5], Keisuke Tajima[1] and Takao Someya[1, 2, 3*]

1 RIKEN Center for Emergent Matter Science (CEMS), Wako, 351-0198 Saitama, Japan

2 Thin-Film Device Laboratory, RIKEN, 2-1 Hirosawa, Wako, 351-0198 Saitama, Japan

3 Department of Electrical Engineering and Information Systems, The University of Tokyo, 113-8656 Tokyo, Japan

4 Institute of Engineering Innovation, The University of Tokyo, 113-8656 Tokyo, Japan

5 Wuhan National Laboratory for Optoelectronics, Huazhong University of Science and Technology, Wuhan, China

[*]e-mail: kenjiro.fukuda@riken.jp; takao.someya@riken.jp

Supplementary Figures

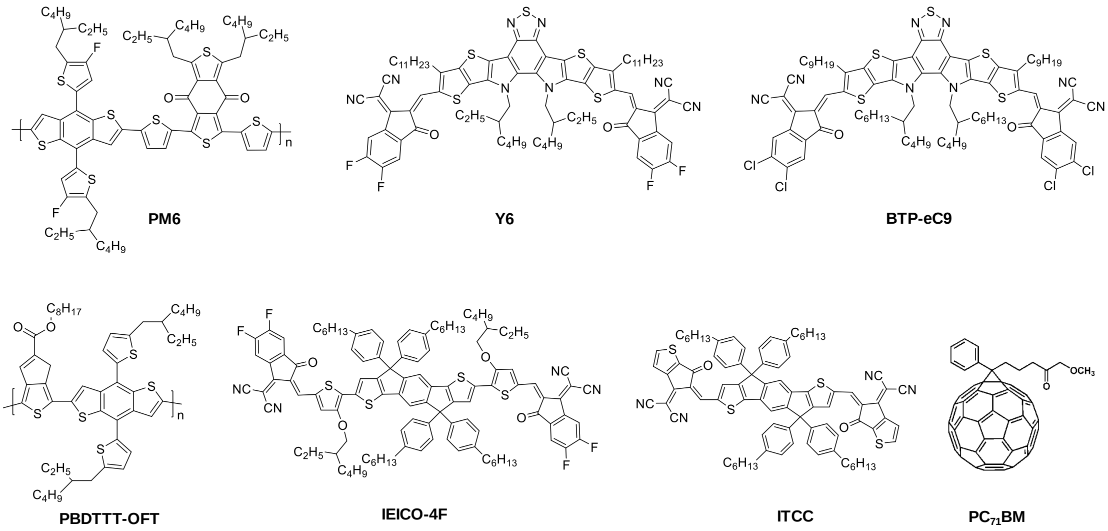

Supplementary Figure 1 Chemical structure of donor and acceptor materials used in this study. Chemical structure of the polymer donors PM6 and PBDTTT-OFT, and acceptors Y6, BTP-eC9, IEICO-4F, ITCC, and $PC_{71}BM$.

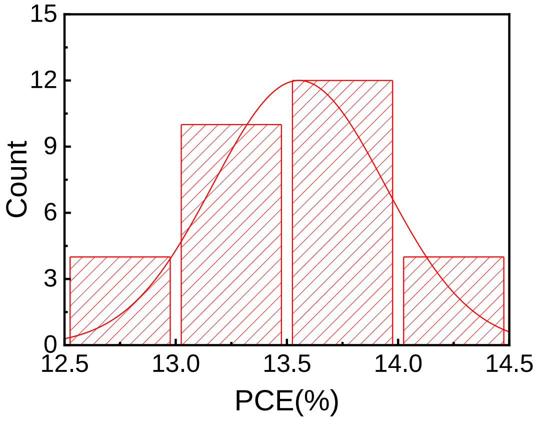

Supplementary Figure 2 Statistical histogram of devices with in-situ HTL growth. Power conversion efficiency (PCE) distribution of 30 devices (tPI/ITO/PEI-Zn/PM6:Y6/AgO_x/Ag/Parylene) after 85 °C annealing treatment for 24 h.

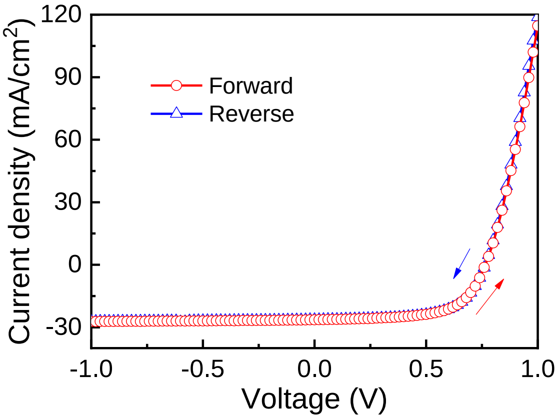

Supplementary Figure 3 Hysteresis of the devices with in-situ growth of HTL. *J*–*V* curves for one of the optimized cells (tPI/ITO/PEI-Zn/PM6:Y6/AgO$_x$/Ag/Parylene) were measured by forward and reverse scans, showing negligible hysteresis.

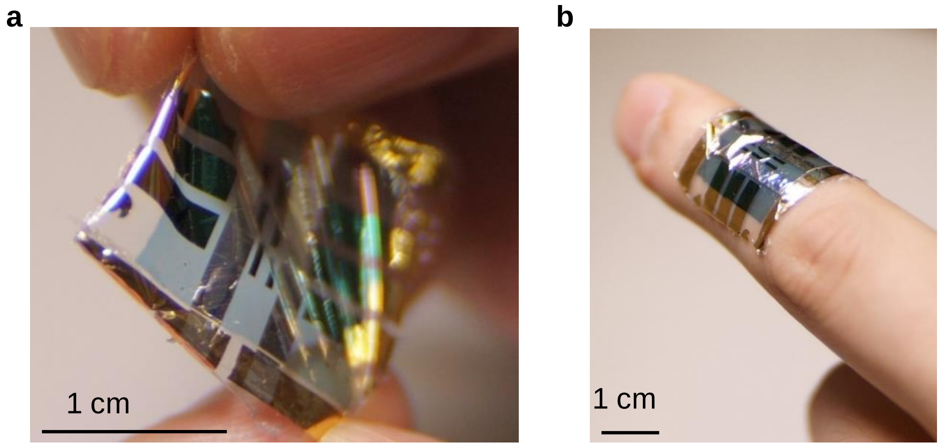

Supplementary Figure 4 Photographs of the free-standing OPVs. a, Photograph of the free-standing OPVs after delamination from the supporting glass substrate. b, Photograph of the free-standing OPVs attached to a finger. The scale bar is 1 cm.

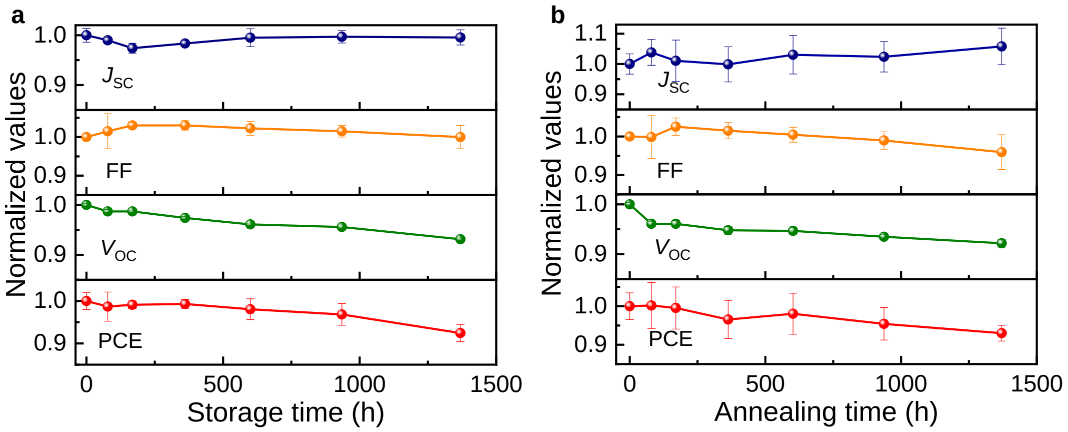

Supplementary Figure 5 Long-term storage stability of free-standing OPVs in air. Normalized values of the short-circuit current density (J_{SC}), fill factor (FF), open-circuit voltage (V_{OC}), and power conversion efficiency (PCE) as a function of storage stability in air for free-standing OPVs (tPI/ITO/PEI-Zn/PM6:Y6/AgO_x/Ag/Parylene): a, at room temperature (20–25 °C) under dark conditions; b, at 85 °C under dark conditions.

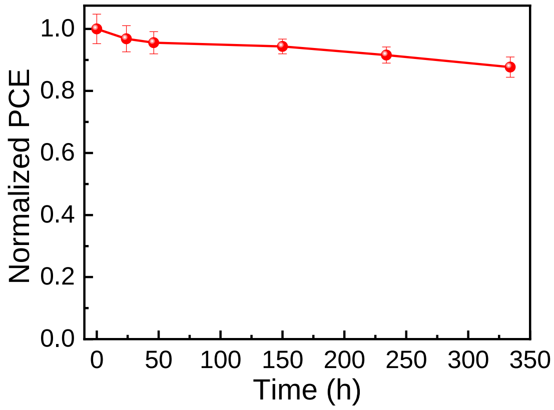

Supplementary Figure 6 Light stability of free-standing OPVs in N_2-filled glovebox. Normalized PCE as a function of light illumination time of devices with structure of tPI/ITO/PEI-Zn/PM6:Y6/AgO_x/Ag/Parylene under AM 1.5G (1-sun) illumination with a filter cutting off light below 400 nm in an N_2-filled glovebox.

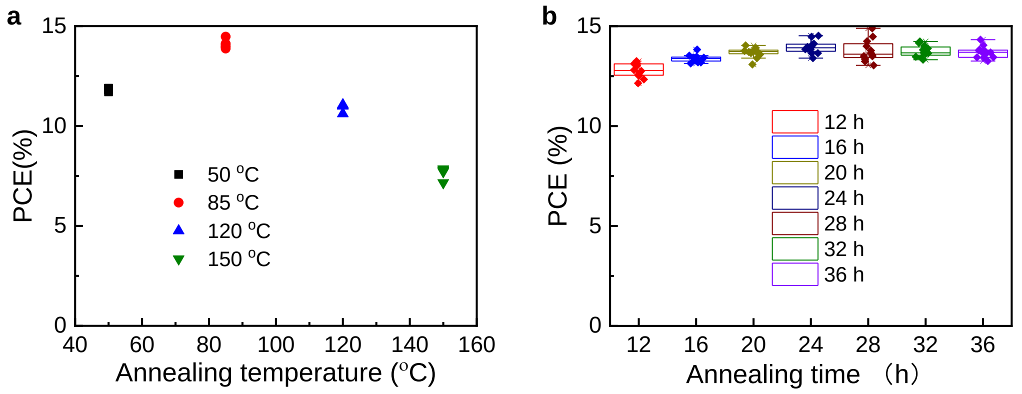

Supplementary Figure 7. Optimization of annealing temperature and time. a, PCE distribution for the devices (tPI/ITO/PEI-Zn/PM6:Y6/AgO_x/Ag/Parylene) under different annealing temperatures for 24 h. b, The evolution of PCE with annealing time (12 h–36 h) at 85 °C in air (tPI/ITO/PEI-Zn/PM6:Y6/AgO_x/Ag/Parylene). These are statistical values of average and standard deviation obtained from 12 samples. The optimal annealing treatment condition is 85 °C.

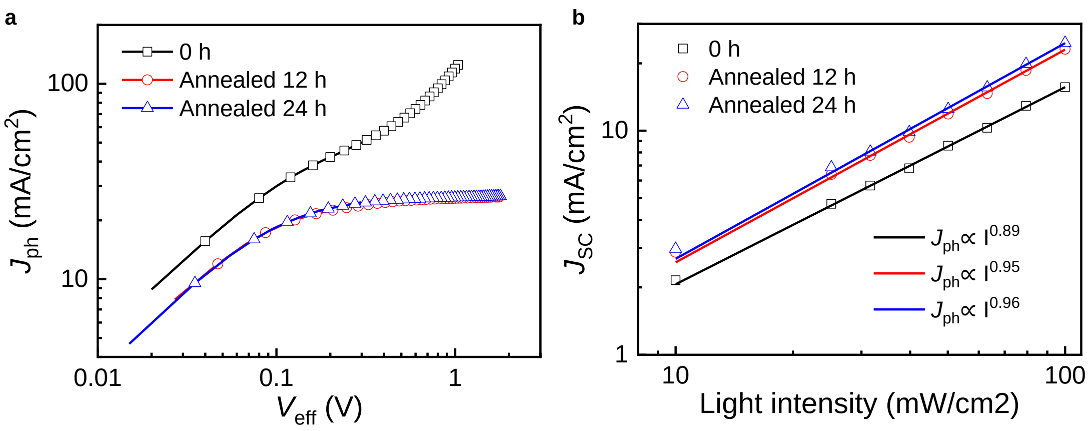

Supplementary Figure 8 Analysis of extraction and transportation of charge carriers. a, The curves of J_{ph} versus V_{eff} in the devices (tPI/ITO/PEI-Zn/PM6:Y6/AgO$_x$/Ag/Parylene) for different annealing times at 85 °C. b, The dependence of P_{light} on J_{SC} of the devices (tPI/ITO/PEI-Zn/PM6:Y6/AgO$_x$/Ag/Parylene) under different annealing times at 85 °C.

Here, $J_{ph} = J_L - J_D$, and $V_{eff} = V_0 - V_A$, where J_L is the current density under illumination and J_D is the current density in darkness, V_0 is the voltage when $J_{ph} = 0$, and V_A is the applied voltage. As shown in Supplementary Figure 4a, the J_{ph} of the initial device cannot achieve a saturated value at low V_{eff}, indicating the poor dissociation of excitons into electrons and holes. In contrast, the J_{ph} of annealed devices is saturated, suggesting high exciton dissociation efficiency in annealed devices. The charge recombination can be investigated by the function of $J_{SC} \propto (P_{light})^{\alpha}$. As presented in Supplementary Figure 4b, the fitting α value in the device with 24 h annealing treatment is 0.96, which is closer to 1 compared to other devices and demonstrates the weaker bimolecular recombination. These results demonstrate that annealing treatment is an effective approach to improve the exciton dissociation efficiency and suppress recombination, thus improving J_{SC} and FF simultaneously.

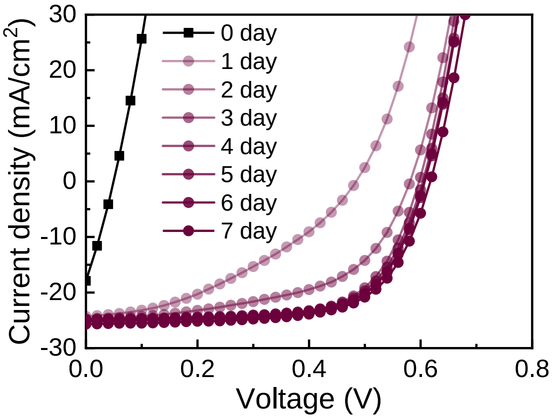

Supplementary Figure 9 PCE evolution of a device under natural oxidation treatments. *J*–*V* curves for OPVs based on PM6:Y6 active layer with Ag electrode (tPI/ITO/PEI-Zn/PM6:Y6/Ag) under natural oxidation treatments. The device is stored in ambient air under room temperature to promote natural oxidation of Ag electrode. The black line is for the initial device and dark red is for oxidized devices. The darker the color, the longer the oxidation time.

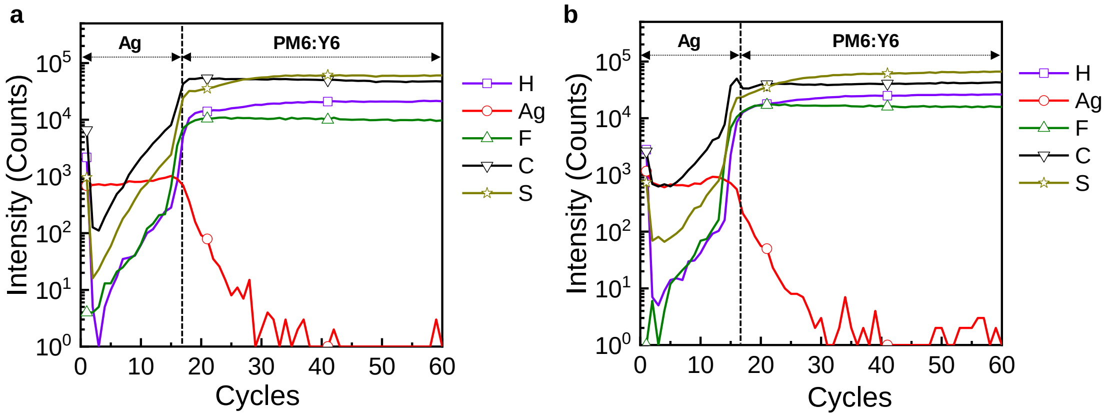

Supplementary Figure 10 Depth profiles of different elements using dynamic secondary ion mass spectrometry (D-SIMS). a, Depth profiles of H, Ag, F, C, and S for the pristine sample with structure of glass/ITO/PEI-Zn/PM6:Y6/Ag. **b**, Depth profiles of H, Ag, F, C, and S for the sample annealed at 85 °C in air for 24 h. The Ag and PM6:Y6 regions can be determined by the Ag signal and F and S signals, respectively.

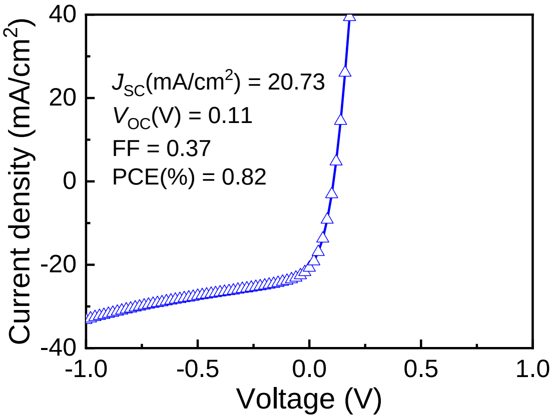

Supplementary Figure 11 Performance of the device annealed in N_2-filled glovebox. *J*–*V* curves for OPVs based on PM6:Y6 active layer with Ag electrode (tPI/ITO/PEI-Zn/PM6:Y6/Ag) annealed in an N_2-filled glovebox at 85 °C for 24 h.

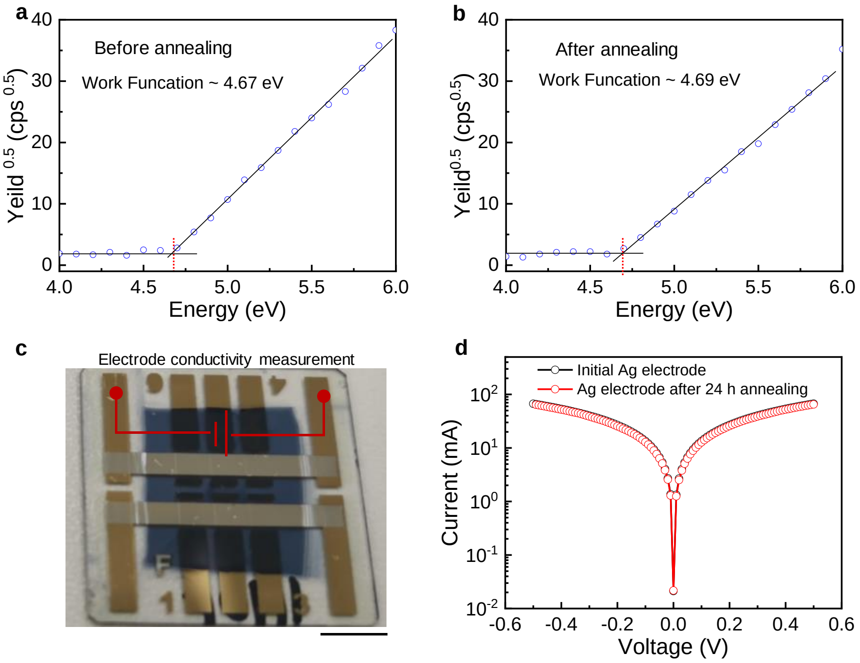

Supplementary Figure 12 Work function and conductivity of the electrode. a, Work function of the top surface of the Ag electrode before annealing. b, Work function of the top surface of the Ag electrode after 24 h annealing at 85 °C in air. c, Demonstration of the conductivity measurement for the common Ag electrode. Scale bar is 5 mm. d, Conductivity of the Ag electrode before and after annealing.

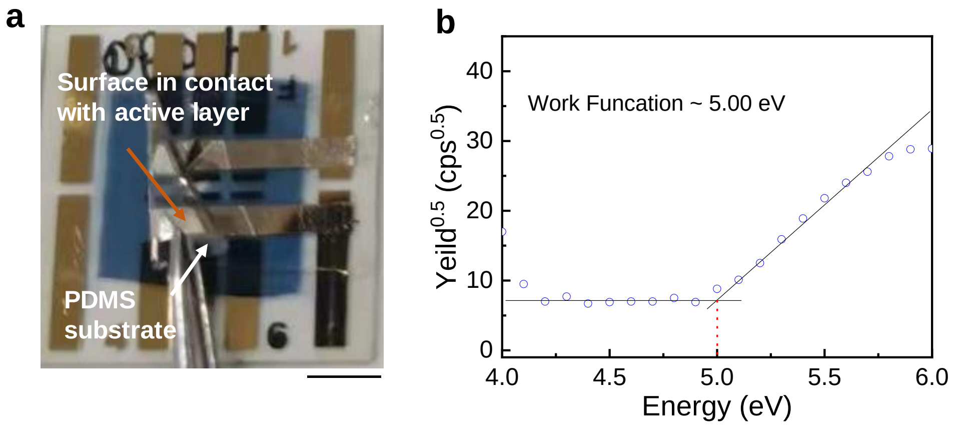

Supplementary Figure 13 Work function measurement. a, Schematic diagram of removeable electrode. Scale bar is 5 mm. b, The device with removeable electrode was annealed in ambient air for 24 h. The removeable electrode was then detached and the work function of the surface in contact with the active layer was measured by using photoemission spectroscopy.

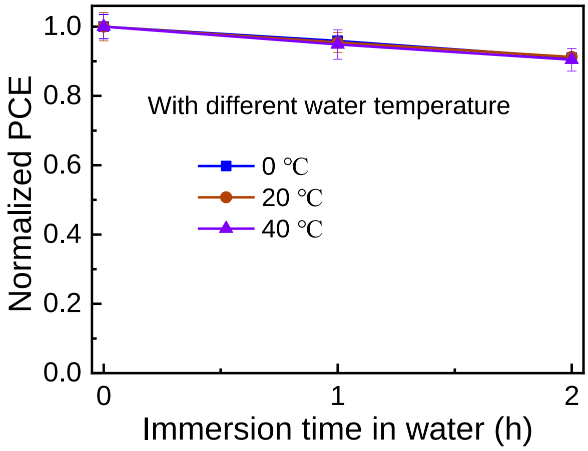

Supplementary Figure 14 Waterproofness of OPVs under different water temperatures. The waterproofness tests of devices with structure tPI/ITO/PEI-Zn/PM6:Y6/AgO_x/Ag/Parylene under different water temperatures.

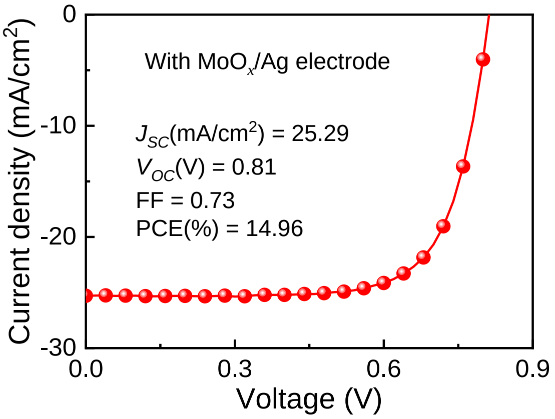

Supplementary Figure 15 Performance of the device with MoO$_x$/Ag electrode. *J*–*V* curves for free-standing OPVs based on PM6:Y6 active layer with conventional MoO$_x$ hole-transporting layer (tPI/ITO/PEI-Zn/PM6:Y6/MoO$_x$/Ag/Parylene).

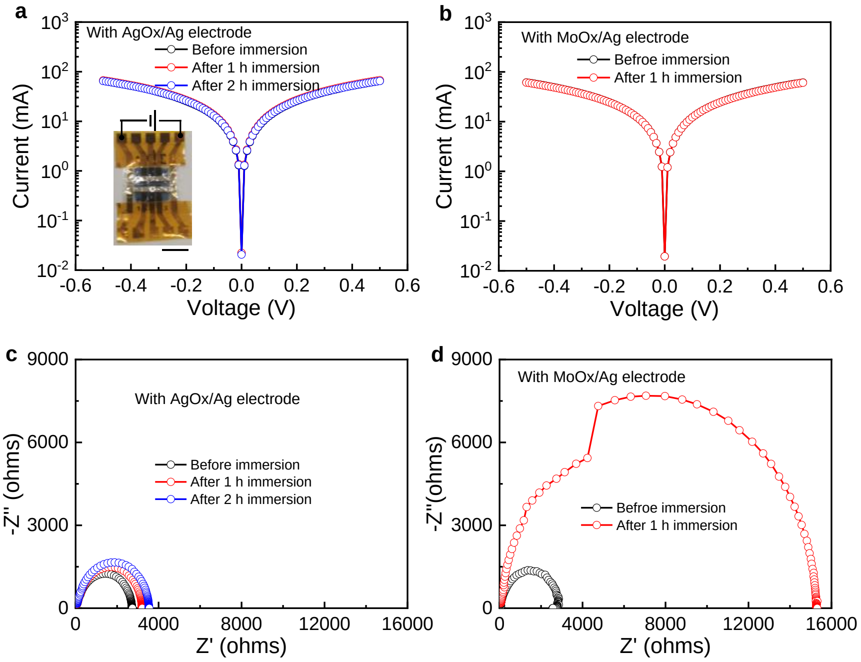

Supplementary Figure 16 Conductivity of electrodes and the impedance spectra of devices. a, Conductivity of AgO_x/Ag electrode before and after water immersion. The inset is the demonstration of the conductivity measurement of the device common electrode. Scale bar is 1 cm. b, Conductivity of the MoO_x/Ag electrode before and after water immersion c, Impedance spectra of devices based on the AgO_x/Ag electrode before and after water immersion. d. Impedance spectra of devices based on the MoO_x/Ag electrode before and after water immersion.

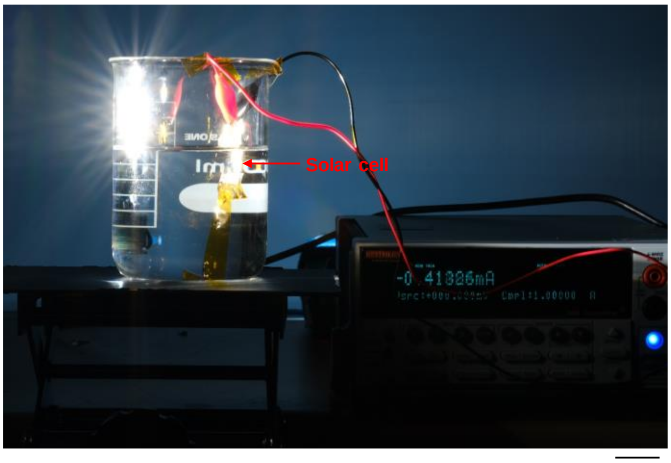

Supplementary Figure 17 Operation test underwater. Photograph of the free-standing OPV operating underwater. Scale bar is 1 cm.

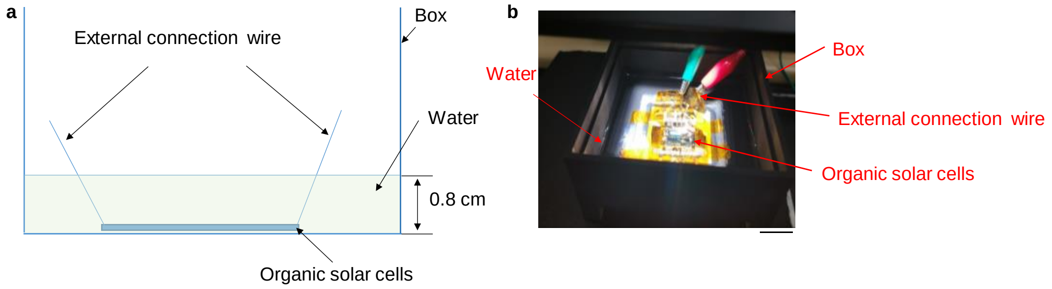

Supplementary Figure 18 Steady-state J_{SC} measurement underwater. a, Schematic diagram of the cross-section of the measurement setup. b, Photograph of setup for the measurement of steady-state current in water. Scale bar is 2 cm.

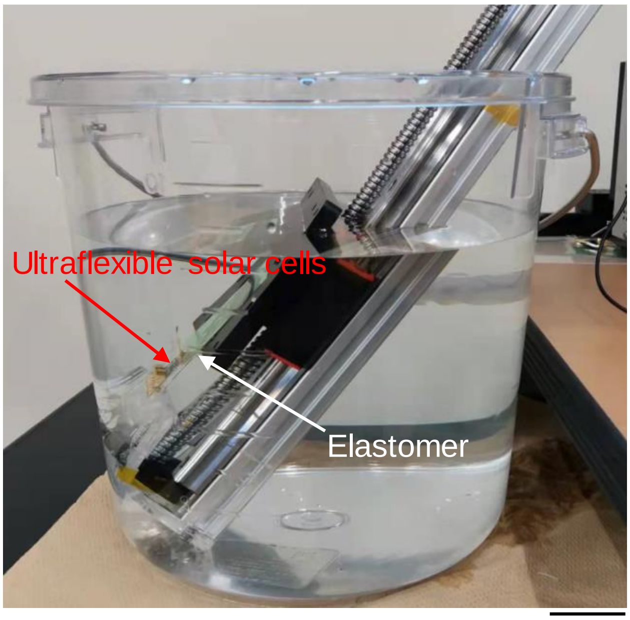

Supplementary Figure 19 Mechanical durability test underwater. Photograph of free-standing OPVs (tPI/ITO/PEI-Zn/PM6:Y6/AgO_x/Ag/Parylene) for stretching–compressing test in water. Scale bar is 1 cm.

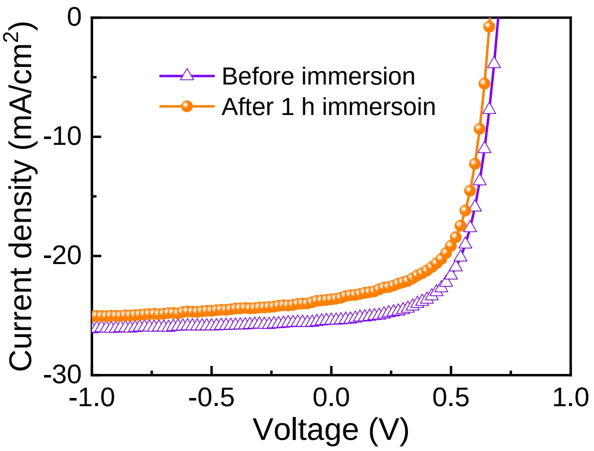

Supplementary Figure 20 Water stability test on naturally oxidized devices. *J–V* curves for OPVs based on PM6:Y6 active layer with naturally oxidized Ag electrode for seven days in ambient air at room temperature (Glass/ITO/PEI-Zn/PM6:Y6/AgO_x/Ag) before and after immersion in water for 1 h. The purple triangle is for the device before immersion. The orange solid sphere is for the devices after 1 h immersion in water.

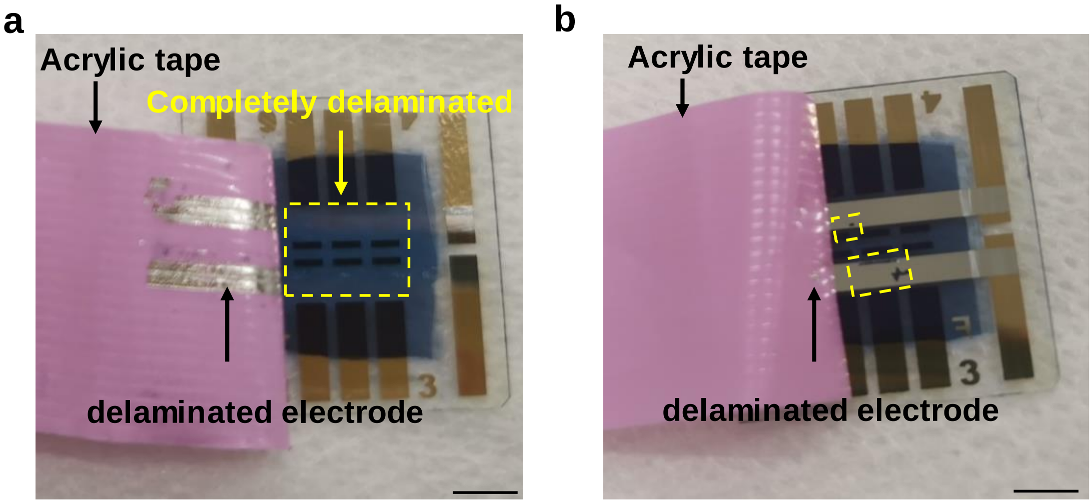

Supplementary Figure 21 Comparison of the adhesion. a, Photograph of the peeling test of the devices under natural oxidation treatment; b, Photograph of the peeling test of the devices under thermal annealing treatment. The scale bar is 5 mm.

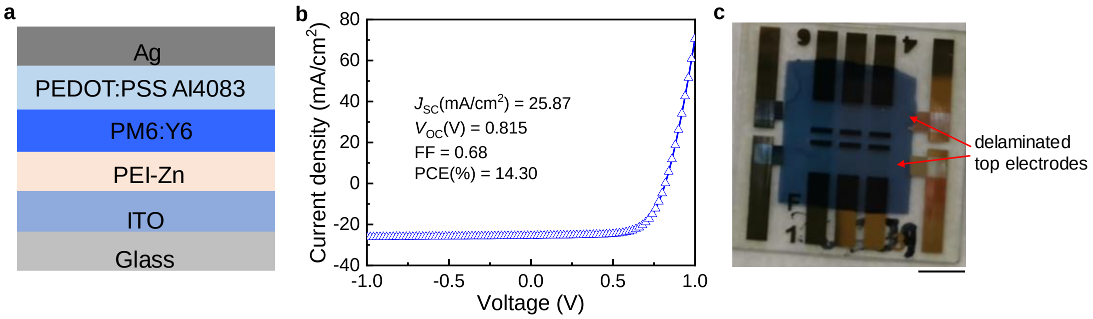

Supplementary Figure 22 Structure and performance of devices based on PEDOT:PSS HTL. a, Device structure with PEDOT:PSS (Clevios AI4083); b, *J–V* characteristics of the device (glass/ITO/PEI-Zn/PM6:Y6/PEDOT:PSS/Ag); c, Photograph of the device after immersion for 1 h in water.

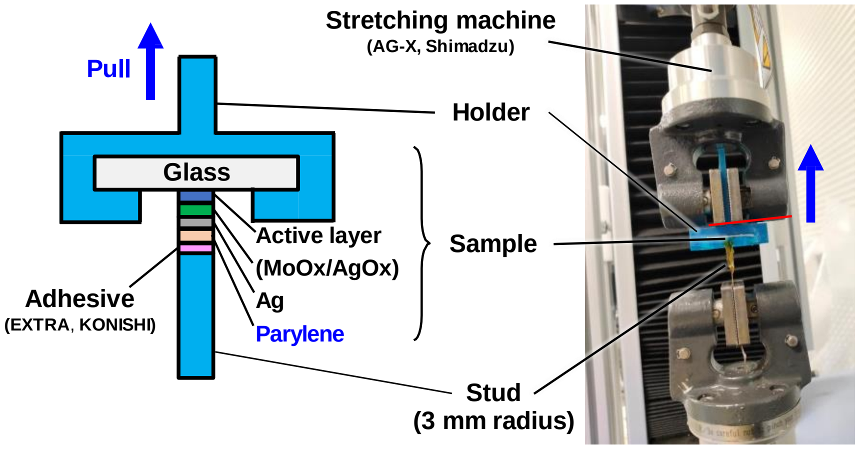

Supplementary Figure 23 Equipment for adhesion measurement. Photograph and schematic diagram for adhesion measurement.

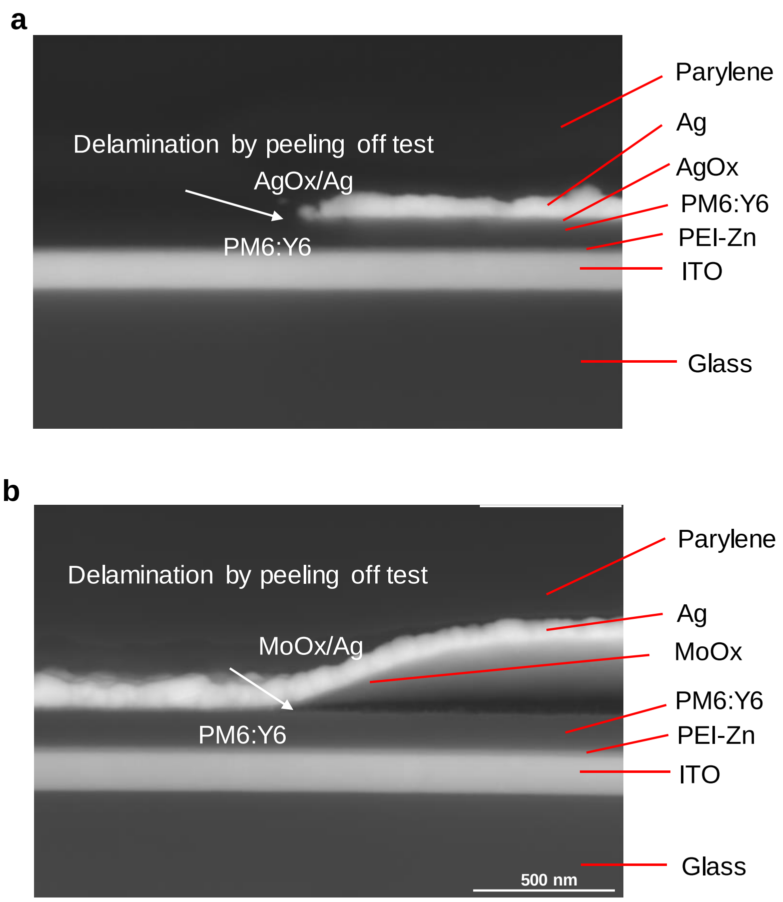

Supplementary Figure 24 Cross-sectional observation of OPVs. Cross-sectional scanning electron microscopy (SEM) images of peeled-off area. A stud was attached to the samples. The stretching machine then clamped the stud and gradually peeled it off from the sample. Cross-sectional SEM was applied to verify the delamination position. a, Sample with structure of glass/ITO/PEI-Zn/PM6:Y6/AgO_x/Ag/Parylene; b, Sample with structure of glass/ITO/PEI-Zn/PM6:Y6/MoO_x/Ag/Parylene. Scale bar is 500 nm.

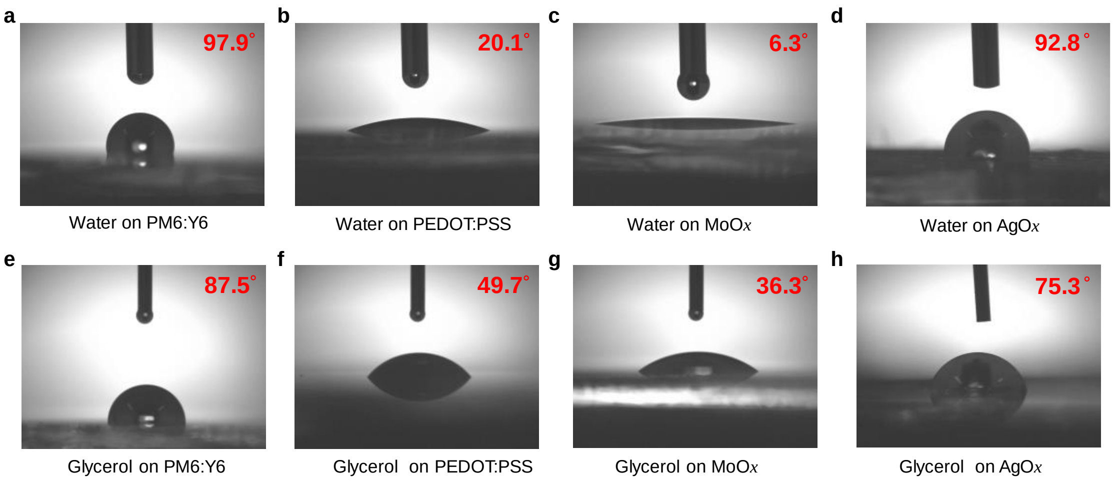

Supplementary Figure 25 Contact angles measurement of active layer and HTL surfaces. Water contact angle images of a, PM6:Y6; b, PEDOT:PSS; c, MoO_x; and d, AgO_x. Glycerol contact angle images of e, PM6:Y6; f, PEDOT:PSS; g, MoO_x; and h, AgO_x.

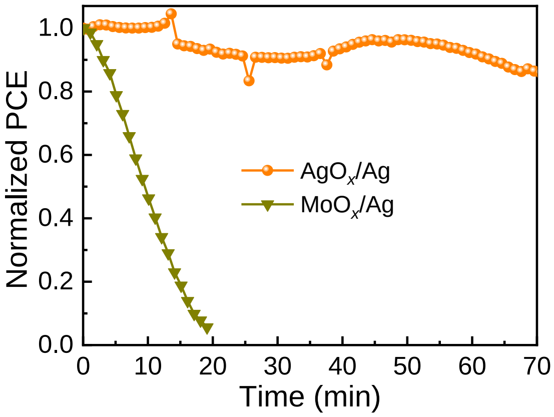

Supplementary Figure 26 Operational stabilities in water. Maximum power point tracking of devices immersed in water with the structure of Parylene/tPI/ITO/PEI-Zn/PM6:Y6/HTLs/Ag/Parylene under approximately light illumination. Note that the light illumination could be different from intensity and spectrum of AM 1.5 (standard1-sun) with the presence of water between the device and light source.

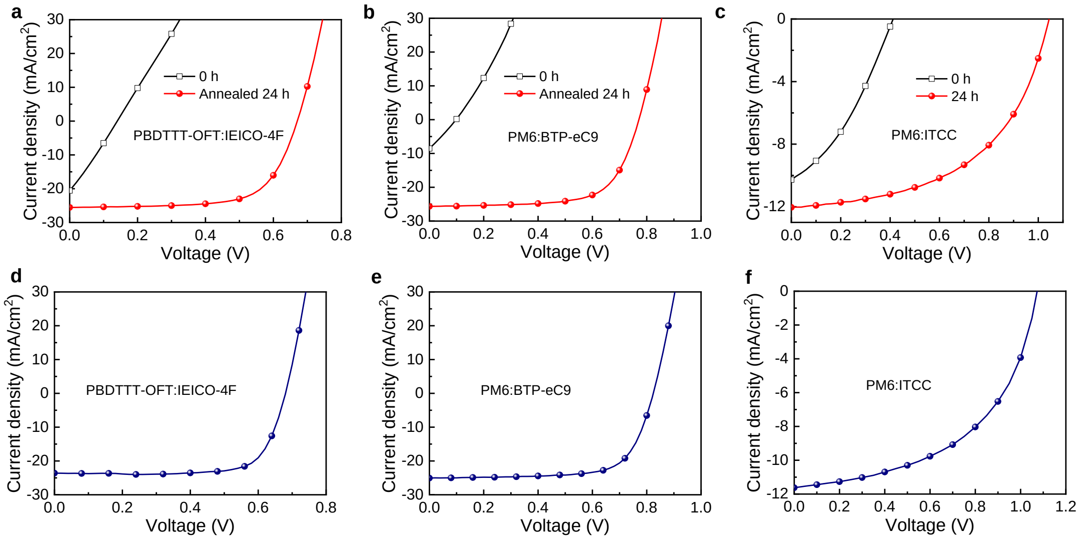

Supplementary Figure 27 Device performance of OPVs with different active layers. *J–V* characteristics of the device with silver (tPI/ITO/PEI-Zn/Active layer/Ag) based on different active layers before and after annealing treatment at 85 °C: a, PBDTTT-OFT:IEICO-4F; b, PM6:BTP-eC9; and c, PM6:ITCC. *J–V* characteristics of the device with MoO_x (tPI/ITO/PEI-Zn/ Active layer /MoO_x/Ag) based on different active layers: d, PBDTTT-OFT:IEICO-4F; e, PM6:BTP-eC9; and f, PM6:ITCC.

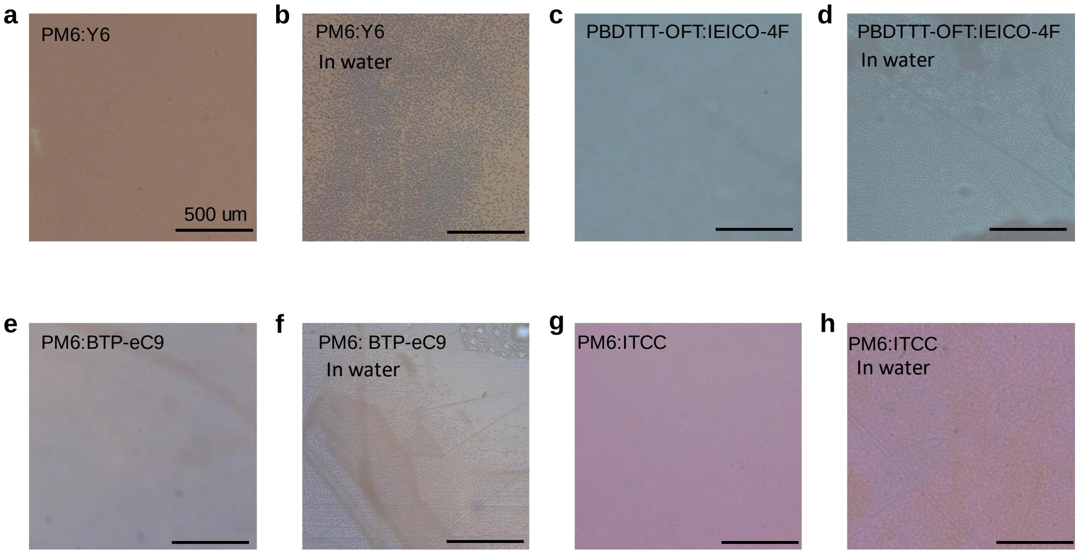

Supplementary Figure 28 Stability of active layers in water. Optical images of active layer samples before and during immersion. a, PM6:Y6 sample before immersion; b, PM6:Y6 sample immersed in water; c, PBDTTT-OFT:IEICO-4F sample before immersion; d, PBDTTT-OFT:IEICO-4F sample immersed in water; e, PM6:BTP-eC9 sample before immersion; f, PM6:BTP-eC9 sample immersed in water; g, PM6:ITCC sample before immersion; and h, PM6:ITCC sample immersed in water. The scale bar is 500 μm.

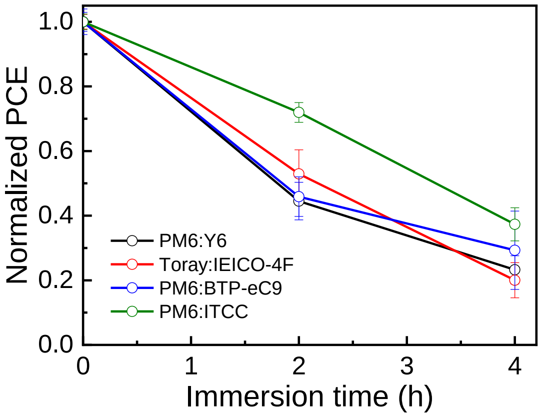

Supplementary Figure 29 Water stability of OPVs with different active layers. The waterproofness measurement of devices with structure of Parylene/tPI/ITO/ZnO/Active layer/MoO_x/Ag/Parylene based on PM6:Y6, PBDTTT-OFT:IEICO-4F, PM6:BTP-eC9, and PM6:ITCC active layer materials.

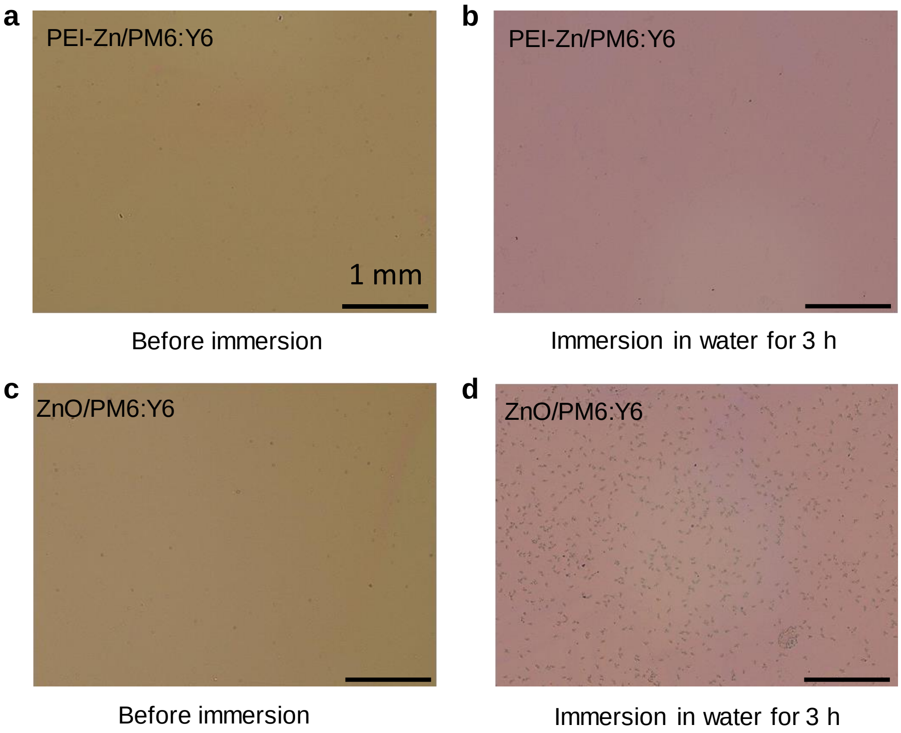

Supplementary Figure 30 Stability in water of PM6:Y6/ETLs. Optical images of bilayer samples before and after immersion. a, PEI-Zn/PM6:Y6 sample before immersion; b, PEI-Zn/PM6:Y6 sample after immersion; c ZnO/PM6:Y6 sample before immersion; d, ZnO/PM6:Y6 sample after immersion.

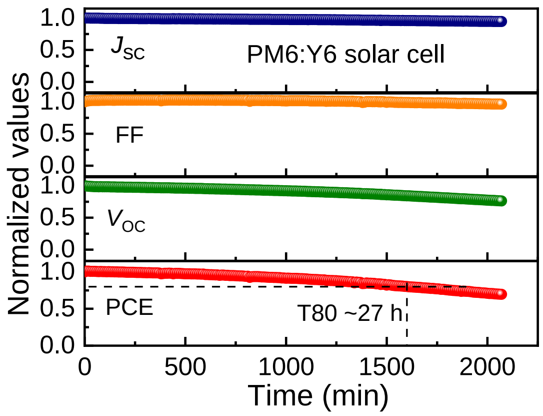

Supplementary Figure 31 Operational stability of OPVs with PM6:Y6. Maximum power point tracking of double-side encapsulated free-standing OPVs (Parylene/tPI/ITO/PEI-Zn/PM6:Y6/AgO*x*/Ag/Parylene) in air under 1-sun illumination.

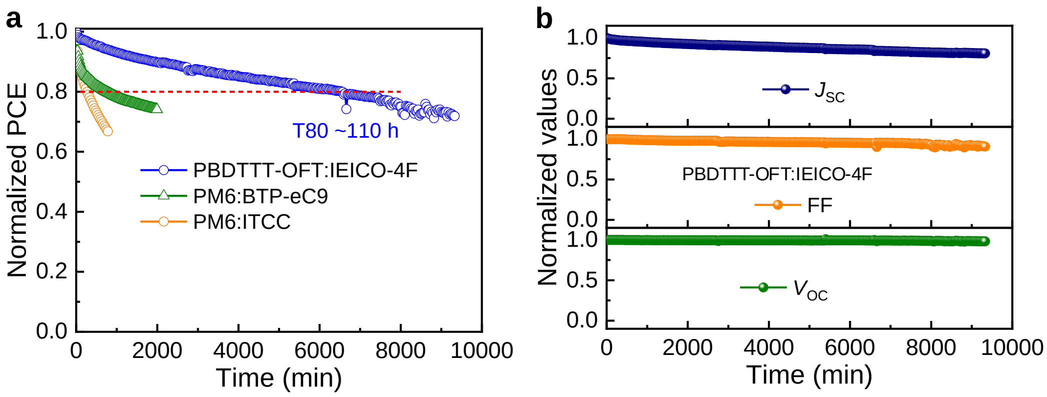

Supplementary Figure 32 Operational stability of OPVs with different active layers. a, Maximum power point tracking of double-side encapsulated free-standing OPVs (Parylene/tPI/ITO/PEI-Zn/Active layers/AgO_x/Ag/Parylene) in air under 1-sun illumination; b, The evolution of J_{SC}, FF, and V_{OC} of double-side encapsulated PBDTTT-OFT:IEICO-4F solar cell.

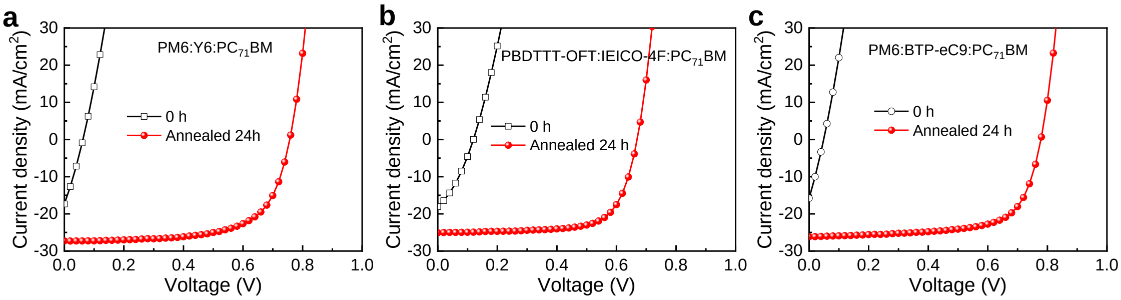

Supplementary Figure 33 Performance evolution of OPVs with ternary blend active layers. The *J*–*V* characteristics of ternary active layer devices with different active layers before and after 85 °C annealing: a, PM6:Y6:$PC_{71}BM$; b, PBDTTT-OFT:IEICO-4F:$PC_{71}BM$; and c, PM6:BTP-eC9:$PC_{71}BM$.

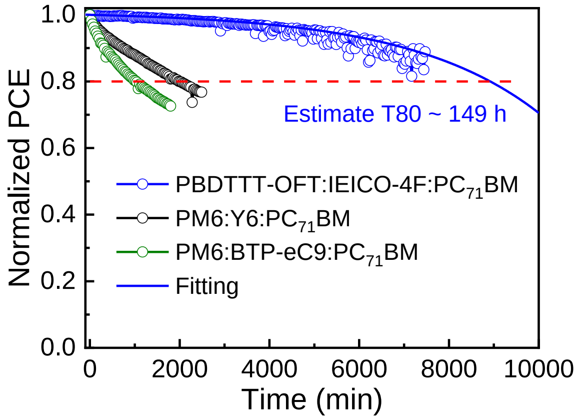

Supplementary Figure 34 Operational stability of OPVs with ternary blend active layers. The operational stability under MPPT in ambient air of devices with structure of Parylene/tPI/ITO/PEI-Zn/Active layer/AgO_x/Ag/Parylene based on PBDTTT-OFT:IEICO-4F:$PC_{71}BM$, PM6:Y6:$PC_{71}BM$, and PM6:BTP-eC9:$PC_{71}BM$ active layer materials. The estimate T80 lifetime of PBDTTT-OFT:IEICO-4F:$PC_{71}BM$ is approximately 149 h.

Supplementary Tables

Supplementary Table 1 Summary of the performance progress of waterproof and wearable OPVs.

Device structure	PCE (%)	Waterproofness test	Remaining (%)	Ref.
Woven fabric/Ag/PEI/P3HT:PC61BM/PH1000	2.9	Wash by water	/	1
Textile substrate/bottom encapsulation barrier/Ag cathode/PFN/PTB7-Th:PC71BM/MoO3/Ag anode/MoO3/top encapsulation barrier	7.27	20 washing cycles at 200 rpm for 10 min duration	99	2
Parylene/ITO/ZnO/PNTz4T:PC71BM/MoOx/Ag/Parylene	7.9	Immersion in water for 120 min	79.2	3
Elastomer/Parylene/ITO/ZnO/PNTz4T:PC71BM/MoOx/Ag/Parylene/Elastomer	7.9	Immersion in water for 120 min	94.6	
Stainless steel wire/PDINO/PM6:Y6:PC71BM/Al 4083/PH1000/CNT wire	9.4	Immersion in water	/	4
tPI/ITO/PEI-Zn/PM6:Y6/Ag/Parylene	14.3	Immersion in water for 4 h	89.6	This work
tPI/ITO/PEI-Zn/PM6:Y6/Ag/Parylene	14.3	Machine washing for 120 min	90	

Supplementary Table 2 Summary of the progress for air stability of flexible OPVs.

Active layer	Anode	Cathode	Initial PCE (%)	Test condition	Time (h)	PCE Retention (%)	Ref.
PTB7-Th: PC71BM	CzPAF-TPA/Ag	ZnO/ITO	6.82	In air at RT	~1800	82	5
PTB7-Th: PC71BM	PEDOT:PSS/Ag	ZnO/ITO	7.52	In air at RT	~1800	67	5
PNTz4T :PC71BM	MoO*x*/Ag	ZnO/ITO	7.9	In air at RT	720	54	3
PV2000	PEDOT: PSS/AgNW	ZnO-NP /Ag-NP	0.4	In air at RT	3600	6.6	6
PBDTTT-OFT :PC71BM	PEDOT :PSS/Ag	ZnO/ITO	10.0	In air at 85 ℃	~500	~80	S7
PBDTTT-OFT:IEICO-4F	MoO_x/Ag	ZnO/ITO	13.2	In air at RT	3194	95.7	8
PBDTTT-OFT :IEICO-4F	MoO_x/Ag	ZnO/ITO	13.2	In air at 85 ℃	1050	80	8
PM6:N3: PC71BM	MoO_3/Ag	ZnO/PPZA	15.5	In air at RT	~ 60	~73	9
PM6:N3: PC71BM	MoO_3/Ag	ZnO/PZA	13.5	In air at RT	~ 60	~77	9
PM6:N3: PC71BM	MoO_3/Ag	ZnO/AgNW	4.2	In air at RT	~ 60	~78	9
PBDB-T -2F:Y6	MoO_3/Ag	ZnO/ITO	12.13	In air at RT	144	61	10
PBDB-T -2F:Y6	MoO_3/Ag	AZO-SG/Em-Ag	15.21	In air at RT	144	60	10
PM6:Y6	MoO_x/Ag	PEI-Zn/ITO	15.8	In air at RT	1574	89.6	11
PM6:Y6	MoO_x/Ag	PEI-Zn/ITO	15.8	In air at 85 ℃	172	92.4	11
PBDB-T-2F: Y6:PC70BM	MoO_x/Ag	c-PEIE/ITO	16.45	In air at RT	576	95.73	12
PBDB-T-2F: Y6:PC70BM	MoO_x/Ag	PEIE/ITO	12.58	In air at RT	576	70.49	12
PM6:Y6	Oxidized Ag	PEI-Zn/ITO	14.3	In air at RT	1369	92.4	This
PM6:Y6	Oxidized Ag	PEI-Zn/ITO	14.3	In air at 85 ℃	1371	93	work

RT: room temperature

Supplementary Table 3. Summary of the light stability progress of flexible OPVs.

Device structure	Thickness	Test conditions	Duration (h)	PCE (%)	Remaining (%)	Ref.
AgNWs@PI/ZnO/PM6:Y6: IDIC/H_xMoO_3/PEDOT:PSS /AgNWs	~80 µm	Light stability in glovebox under LED illumination	150 h	11.9	~79	13
PEN/AgNWs/PEI-Zn/PM6:Y6/MoO_x/Ag/Al2 O3 encapsulation	~2 µm	Light stability in glovebox under 1-sun with 400 nm filter	200	15.0	>80	14
PET/PH1000/PEDOT: PSS (4083)/active layer/PDINO/ultrathin metal	/	Light stability under 1-Sun irradiation (atmosphere is not specified)	2.5	15.1	95	15
PET/hc-PEDOT:PSS/ZnO/PBDB-T:ITIC/MoO_3/Ag	/	MPPT (atmosphere is not specified)	0.33	7.58	/	16
PET/hc-PEDOT:PSS/ZnO/PTB7-Th:PC71BM /MoO_3/Ag	/	MPPT (atmosphere is not specified)	0.33	8.9	/	16
Parylene/ITO/PEI-Zn/PM6:Y6/MoO_x/Ag/Paryl ene	~3 µm	MPPT in air	0.16	14.03	<70	17
tPI/ITO/PEI-Zn/PM6:Y6/MoO_x/Ag/Paryl ene	~3 µm	MPPT in air	25	15.8	77.1	11
tPI/ITO/PEI-Zn/PM6:Y6/AgO_x/Ag/Paryl ene	~3 µm	Light stability in glovebox under 1-sun with 400 nm filter	334	14.3	87.7	This work
Parylene/tPI/ITO/PEI-Zn/PBDTTT-OFT:IEICO-4F/ AgO_x/Ag /Parylene	~4 µm	MPPT in air	110	11.9	80	This work
Parylene/tPI/ITO/PEI-Zn/PBDTTT-OFT:IEICO-4F:$PC_{71}BM$/ AgO_x/Ag /Parylene	~4 µm	MPPT in air	149	11.9	80	This work

Supplementary Table 4. Average photovoltaic parameters of ultraflexible OPVs annealing at 85 °C in air from 12 h to 36 h.

Annealing time (h)	J_{SC} (mA cm^{-2})	V_{OC} (V)	FF	PCE (%)
12	24.0 ± 1.2	0.76 ± 0.01	0.70 ± 0.03	12.8 ± 0.3
16	25.1 ± 0.2	0.76 ± 0.01	0.70 ± 0.01	13.4 ± 0.2
20	24.9 ± 0.3	0.77 ± 0.01	0.71 ± 0.01	13.7 ± 0.2
24	25.4 ± 0.4	0.78 ±0.01	0.70 ± 0.01	13.9 ± 0.3
28	24.6 ± 0.5	0.79 ±0.01	0.71 ± 0.01	13.8 ± 0.5
32	24.7 ± 0.2	0.79 ± 0.01	0.70 ± 0.01	13.7 ± 0.3
36	24.7 ± 0.3	0.79 ± 0.01	0.70 ±0.01	13.7 ± 0.3

These are statistical values of average and standard deviation obtained from 12 samples.

Supplementary Table 5. Average photovoltaic parameters of ultraflexible OPVs stored in air at room temperature under dark for natural oxidization for 0–7 days.

Storage time (days)	J_{SC} (mA cm^{-2})	V_{OC} (V)	FF	PCE (%)
0	16.70 ± 0.72	0.04 ± 0.01	0.27 ± 0.01	0.18 ± 0.03
1	23.93 ± 0.64	0.38 ± 0.17	0.43 ± 0.10	4.29 ± 2.88
2	24.65 ± 0.37	0.58 ± 0.03	0.58 ± 0.11	8.32 ± 2.04
3	24.91 ± 0.46	0.60 ± 0.01	0.67 ± 0.01	10.01 ± 0.34
4	24.80 ± 0.33	0.60 ±0.01	0.67 ±0.01	10.06 ± 0.15
5	24.89 ± 0.28	0.60 ±0.01	0.67 ±0.01	10.09 ± 0.10
6	25.24 ± 0.39	0.60 ± 0.01	0.67 ± 0.01	10.25 ± 0.19
7	25.10 ± 0.32	0.61 ± 0.01	0.67 ± 0.01	10.32 ± 0.13

These are statistical values of average and standard deviation obtained from 12 samples.

Supplementary Table 6. Average photovoltaic parameters of OPVs after seven days of natural oxidation before and after 1 h immersion in water.

Situation	J_{SC} (mA cm^{-2})	V_{OC} (V)	FF	PCE (%)
Before immersion	25.09 ± 0.29	0.69 ± 0.01	0.60 ± 0.02	10.32 ± 0.43
After 1 h of immersion	23.40 ± 0.47	0.68 ± 0.01	0.58 ± 0.03	9.20 ± 0.45

These are statistical values of average and standard deviation obtained from six samples.

Supplementary Table 7. Summary of the surface energies of PM6:Y6, PEDOT:PSS, MoO_x, and AgO_x and thermodynamic work of adhesions between PM6:Y6 and different HTLs.

Materials	γ^d (mJ/m^2)	γ^p(mJ/m^2)	γ(mJ/m^2)	W_a(mJ/m^2)
PM6:Y6	14.84	3.52	17.63	—
PEDOT:PSS	0.27	91.06	91.33	14.62
MoO_x	3.41	80.21	83.62	24.55
AgO_x	29.67	1.67	31.34	44.08

Supplementary Table 8 Photovoltaic parameters of OPVs based on the different active layers under 100 mW cm^{-2} AM 1.5 G illumination.

Active layer	Electrode	Treatment	J_{SC} (mA cm^{-2})	V_{OC} (V)	FF	PCE (%)
PBDTTT-OFT:IEICO-4F	Ag	Without treatment	20.6	0.17	0.27	1.0
		85 °C for 24 h	25.6	0.67	0.68	11.6
	MoO_x/Ag	Without treatment	23.6	0.68	0.75	12.1
PM6:BTP-eC9	Ag	Without treatment	13.3	0.09	0.25	0.3
		85 °C for 24 h	25.7	0.77	0.68	13.4
	MoO_x/Ag	Without treatment	25.0	0.82	0.72	14.8
PM6:ITCC	Ag	Without treatment	10.7	0.41	0.37	1.6
		85 °C for 24 h	12.0	1.04	0.52	6.6
	MoO_x/Ag	Without treatment	11.5	1.07	0.52	6.4

References

1. Zhen H, *et al.* Water-borne foldable polymer solar cells: one-step transferring free-standing polymer films onto woven fabric electrodes. *J. Mater. Chem. A* **5**, 782-788 (2017).
2. Jeong EG, Jeon Y, Cho SH, Choi KC. Textile-based washable polymer solar cells for optoelectronic modules: toward self-powered smart clothing. *Energy Environ. Sci.* **12**, 1878-1889 (2019).
3. Jinno H, *et al.* Stretchable and waterproof elastomer-coated organic photovoltaics for washable electronic textile applications. *Nat. Energy* **2**, 780-785 (2017).
4. Lv D, Jiang Q, Shang Y, Liu D. Highly efficient fiber-shaped organic solar cells toward wearable flexible electronics. *npj Flex. Electron.* **6**, 38 (2022).
5. Reddy SS, *et al.* Highly efficient air-stable/hysteresis-free flexible inverted-type planar perovskite and organic solar cells employing a small molecular organic hole transporting material. *Nano Energy* **41**, 10-17 (2017).
6. Li Y, *et al.* Encapsulated Textile Organic Solar Cells Fabricated by Spray Coating. *ChemistrySelect* **4**, 407-412 (2019).
7. Xu X, *et al.* Thermally stable, highly efficient, ultraflexible organic photovoltaics. *Proc. Natl. Acad. Sci. USA* **115**, 4589-4594 (2018).
8. Jiang Z, *et al.* Highly efficient organic photovoltaics with enhanced stability through the formation of doping-induced stable interfaces. *Proc. Natl. Acad. Sci. USA* **117**, 6391-6397 (2020).
9. Qu T-Y, *et al.* Biomimetic Electrodes for Flexible Organic Solar Cells with Efficiencies over 16%. *Adv. Opt. Mater.* **8**, 2000669 (2020).
10. Chen X, *et al.* Realizing Ultrahigh Mechanical Flexibility and >15% Efficiency of Flexible Organic Solar Cells via a "Welding" Flexible Transparent Electrode. *Adv. Mater.* **32**, 1908478 (2020).
11. Xiong S, *et al.* Ultrathin and Efficient Organic Photovoltaics with Enhanced Air Stability by Suppression of Zinc Element Diffusion. *Adv. Sci.* **9**, 2105288 (2022).
12. Jahandar M, *et al.* Highly efficient flexible organic photovoltaic modules for sustainable energy harvesting under low-light condition via suppressing voltage-drop by metal-mediated cross-linkable polymer interfacial layer. *Chem. Eng. J.* **448**, 137555 (2022).
13. Sun L, *et al.* Flexible All-Solution-Processed Organic Solar Cells with High-Performance Nonfullerene Active Layers. *Adv. Mater.* **32**, 1907840 (2020).
14. Qin F, *et al.* Robust metal ion-chelated polymer interfacial layer for ultraflexible non-fullerene organic solar cells. *Nat. Commun.* **11**, 4508 (2020).
15. Song W, *et al.* Ultra-flexible light-permeable organic solar cells for the herbal photosynthetic growth. *Nano Energy* **86**, 106044 (2021).
16. Meng X, *et al.* A General Approach for Lab-to-Manufacturing Translation on Flexible Organic Solar Cells. *Adv. Mater.* **31**, 1903649 (2019).
17. Cheng S, *et al.* Ultrathin Hydrogel Films toward Breathable Skin-Integrated Electronics. *Adv. Mater.* **35**, 2206793 (2023).
