# Supplementary material for: Waterproof and ultraflexible organic photovoltaics with improved interface adhesion
Source: Nat Commun. 2024 Feb 1;15:681. doi: 10.1038/s41467-024-44878-z (PMC10834485; doi:10.1038/s41467-024-44878-z)
Supplement: Supplementary file 3 — Solar Cells Reporting Summary [file 41467_2024_44878_MOESM3_ESM.pdf]

nature research

Corresponding author(s): Kenjiro Fukuda, Takao Someya

Solar Cells Reporting Summary

Nature Research wishes to improve the reproducibility of the work that we publish. This form is intended for publication with all accepted papers reporting the characterization of photovoltaic devices and provides structure for consistency and transparency in reporting. Some list items might not apply to an individual manuscript, but all fields must be completed for clarity.

For further information on Nature Research policies, including our data availability policy, see Authors & Referees.

Experimental design

Please check: are the following details reported in the manuscript?

1. Dimensions

Item	Yes/No	Details
Area of the tested solar cells	☒ Yes ☐ No	Described in "Methods" section. The area is 4 mm^2.
Method used to determine the device area	☒ Yes ☐ No	The device area is defined by the overlap of the ITO electrode and metal electrode.

2. Current-voltage characterization

Item	Yes/No	Details
Current density-voltage (J-V) plots in both forward and backward direction	☒ Yes ☐ No	J-V plots in forward and backward direction are shown in Supplementary Figure 3.
Voltage scan conditions<br>*For instance: scan direction, speed, dwell times*	☒ Yes ☐ No	The solar cells are measured in forward direction.
Test environment<br>*For instance: characterization temperature, in air or in glove box*	☒ Yes ☐ No	Described in"Methods" section. The devices were characterized at room temperature (ca. 20-25°C) in air.
Protocol for preconditioning of the device before its characterization	☐ Yes ☒ No	No preconditioning of the device before its characterization.
Stability of the J-V characteristic<br>*Verified with time evolution of the maximum power point or with the photocurrent at maximum power point; see ref. 7 for details.*	☒ Yes ☐ No	Long-term operational stability tracking at maximum power point under 1-sun light irradiation in air is provided in Supplementary Figure 31 and 32.

3. Hysteresis or any other unusual behaviour

Item	Yes/No	Details
Description of the unusual behaviour observed during the characterization	☐ Yes ☒ No	No hysteresis or other unusual behavior was observed during the characterization.
Related experimental data	☐ Yes ☒ No	N/A

4. Efficiency

Item	Yes/No	Details
External quantum efficiency (EQE) or incident photons to current efficiency (IPCE)	☒ Yes ☐ No	EQE spectra of cells are shown in Figure 1c.
A comparison between the integrated response under the standard reference spectrum and the response measure under the simulator	☒ Yes ☐ No	Integrated EQE under AM1.5 is comparable to current density under the 1-sun simulator illumination.
For tandem solar cells, the bias illumination and bias voltage used for each subcell	☐ Yes ☒ No	No tandem cells are reported in the work.

5. Calibration

Item	Yes/No	Details
Light source and reference cell or sensor used for the characterization	☒ Yes ☐ No	Described in "Methods" section. The light source is XES-40S3, SAN-EI ELECTRIC.
Confirmation that the reference cell was calibrated and certified	☒ Yes ☐ No	Described in "Methods" section. The reference diode is BS-520BK Bunkoukeiki.

Calculation of spectral mismatch between the reference cell and the devices under test

- [ ] Yes
- [x] No

Mismatch is not calculated.

6. Mask/aperture

Size of the mask/aperture used during testing

- [ ] Yes
- [x] No

We did not use mask during testing and the effective area was defined by its crossed area of ITO and metal electrodes.

Variation of the measured short-circuit current density with the mask/aperture area

- [ ] Yes
- [x] No

We did not use a mask during testing.

7. Performance certification

Identity of the independent certification laboratory that confirmed the photovoltaic performance

- [ ] Yes
- [x] No

This work mainly focus on the waterproofness of the devices. We have not certified the efficiency by the independent laboratory.

A copy of any certificate(s)
Provide in Supplementary Information

- [ ] Yes
- [x] No

No certification.

8. Statistics

Number of solar cells tested

- [x] Yes
- [ ] No

Described in Supplementary Figure 2.

Statistical analysis of the device performance

- [x] Yes
- [ ] No

Statistical analysis are shown in Supplementary Figure 2.

9. Long-term stability analysis

Type of analysis, bias conditions and environmental conditions
For instance: illumination type, temperature, atmosphere humidity, encapsulation method, preconditioning temperature

- [x] Yes
- [ ] No

The long-term stability are shown in Supplementary Figures 5 and 6.
